# The GLR-1 phenotypes of the *daf-7(e1372)* allele are not temperature sensitive

**DOI:** 10.17912/micropub.biology.000158

**Published:** 2019-08-26

**Authors:** Annette McGehee

**Affiliations:** 1 Biology Department, Suffolk University, Boston, MA

**Figure 1:**
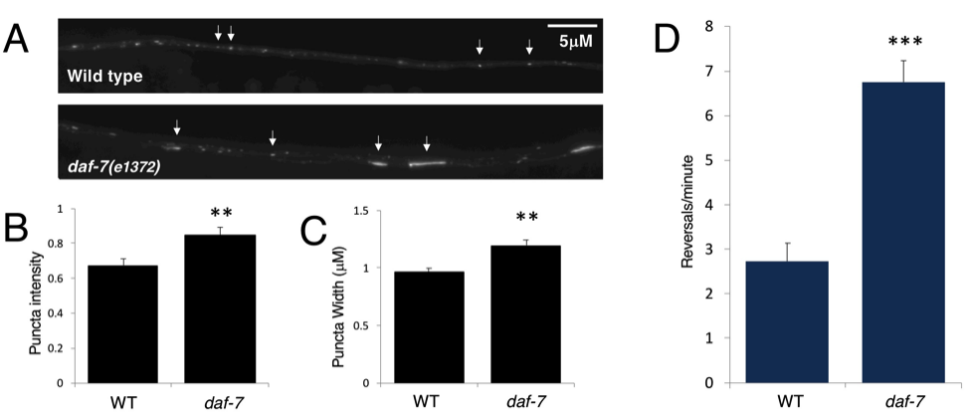
The GLR-1 phenotypes of the *daf-7(e1372)* mutant are present at 15°C. Experiments were performed with young adults that were maintained at 15°C. (A-C) Imaging and quantification of GLR-1::GFP in the ventral nerve cord (VNC) of wild type and *daf-7(e1372*) mutants (n=27 images for each genotype). (A) Representative images of GLR-1::GFP in the VNC of wild type and *daf-7(e1372)* mutants. A subset of the GLR-1::GFP puncta are indicated with white arrows. (B) Quantification of GLR-1::GFP puncta intensity, p=0.008. (C) Quantification of GLR-1::GFP puncta width, p=0.002. (D) Average number of spontaneous reversals per minute for wild type and *daf-7(e1372)* mutants (n=11 for each genotype), p=0.000003. Error bars denote SEM, **p<0.01, ***p<0.001 (Student’s t-test).

## Description

The DAF-7/TGF-β signaling pathway plays a developmental role in the dauer decision; mutants in this signaling pathway have temperature-sensitive constitutive dauer phenotypes (Golden and Riddle 1984, Thomas*et al.* 1993). Additionally, the DAF-7/TGF-β signaling pathway acts in later developmental stages as an environmental sensor to regulate several aspects of metabolism and behavior in response to environmental cues (Greer *et al.* 2008, Guimenny and Savage-Dunn 2013, Meisel *et al.* 2014, Fletcher and Kim 2017). Recently it was shown that the DAF-7/TGF-β signaling pathway is required for normal regulation of levels of the glutamate receptor GLR-1 (McGehee *et al.* 2015). The effect of the DAF-7/TGF-β signaling pathway on GLR-1 was determined both directly using GFP-tagged GLR-1 protein (GLR-1::GFP) and indirectly by measuring the frequency of spontaneous reversals, a behavior that is controlled by signaling through GLR-1 (Zheng *et al.* 1999). The *daf-7(e1372)* allele is described as a temperature-sensitive allele (Riddle *et al.* 1997). When this strain is grown at 15°C worms progress normally through development, and when the growth temperature is shifted to 25°C worms enter the dauer stage (Golden and Riddle 1984). Experiments investigating the effect of the DAF-7/TGF-β signaling pathway on GLR-1 were conducted by growing animals at 15°C, followed by growth at 25°C for several hours prior to performing experiments (McGehee *et al.* 2015). This method of growing the worms at a low temperature and then shifting to a higher temperature is frequently used in studies using DAF-7/TGF-β signaling pathway mutants. However, some of the phenotypes associated with these mutants are not temperature sensitive, for example egg laying (Trent *et al.* 1983), decreased pumping rate and increased fat deposition (Greer *et al.* 2008). These observations suggest that it is the dauer phenotype that is temperature sensitive and not necessarily the *daf-7(e1372)* allele.

To test whether the DAF-7/TGF-β-dependent regulation of GLR-1 is temperature sensitive, animals were grown at 15°C and young adults were examined directly from 15°C cultivation (i.e. without a prior temperature shift to 25°C). Quantitative fluorescence microscopy and spontaneous reversals behavioral assays were performed as previously described (McGehee et al. 2015). The ventral nerve cord (VNC) of young adults expressing a GLR-1::GFP transgene (*nuIs24* (Rongo *et al.* 1998)) was imaged (WT n=27, *daf-7(e1372)* n=27) (Figure 1A). Quanitification of images was done using MetaMorph software to generate linescans of the VNC which were analyzed in IgorPro using custom written software (Burbea *et al.* 2002) to quantify all of the puncta for each worm. There is variability in the puncta widths and intensities that are measured within each image, as can be seen in Figure 1A, so the average intensity and width for all of the puncta in each image was used for comparisons between the genotypes. There was a significant increase in both the intensity (p=0.002) (Figure 1B) and width (p=0.008) (Figure 1C) of GLR-1::GFP puncta in the *daf-7(e1372)* mutants as compared to wild type, indicating that there is more GLR-1::GFP in the *daf-7(e1372)* mutants. Similarly, the rate of spontaneous reversals is increased in *daf-7(e1372)* mutants maintained at 15°C as compared to wild type maintained at 15°C (n=11 of each genotype, p=0.000003). These results show that the previously reported GLR-1 phenotypes, namely the increased GLR-1 levels and spontaneous reversals phenotypes, are not temperature sensitive. Although it is possible that there is a secondary mutation in the *daf-7(e1372)* strain that causes the observed GLR-1 phenotypes, this is unlikely because the GLR-1 phenotypes are identical to those observed in mutants in other components of the DAF-7/TGF-b signaling pathway (McGehee *et al.* 2015). However, since transgenic rescue experiments and/or testing of a second *daf-7* allele have not been performed, the presence of a secondary mutation cannot be formally ruled out. The presence of the GLR-1 phenotypes in the *daf-7(e1372)* strain at low temperature (15°C) is important to note as it supports the idea that it is the dauer phenotype that is temperature-sensitive and not necessarily the *daf-7(e1372)* allele. One implication of this finding is that it is reasonable to study the DAF-7/TGF-β-dependent control of the GLR-1 protein using the *daf-7(e1372)*strain in animals that have been reared at 15°C and not exposed to a temperature shift to 25°C. Additionally, caution should be used when interpreting phenotypes in this strain, and the temperature dependence of novel phenotypes should be determined.

## Reagents

**Table:** 

**Strain name**	**Genotype**	**Available from the CGC**
N2		Yes
CB1372	*daf-7(e1372)III*	Yes
KP1147	*nuIs24(Pglr-1::glr-1::gfp)IV*	No (Rongo et al. 1998)
KP3079	*daf-7(e1372)III; nuIs24(Pglr-1:glr-1::gfp)IV*	No (McGehee et al. 2015)

## References

[R1] Burbea M, Dreier L, Dittman JS, Grunwald ME, Kaplan JM (2002). Ubiquitin and AP180 regulate the abundance of GLR-1 glutamate receptors at postsynaptic elements in C. elegans.. Neuron.

[R2] Fletcher M, Kim DH (2017). Age-Dependent Neuroendocrine Signaling from Sensory Neurons Modulates the Effect of Dietary Restriction on Longevity of Caenorhabditis elegans.. PLoS Genet.

[R3] Golden JW, Riddle DL (1984). A pheromone-induced developmental switch in Caenorhabditis elegans: Temperature-sensitive mutants reveal a wild-type temperature-dependent process.. Proc Natl Acad Sci U S A.

[R4] Greer ER, Pérez CL, Van Gilst MR, Lee BH, Ashrafi K (2008). Neural and molecular dissection of a C. elegans sensory circuit that regulates fat and feeding.. Cell Metab.

[R5] Gumienny TL, Savage-Dunn C (2013). TGF-β signaling in C. elegans.. WormBook.

[R6] McGehee AM, Moss BJ, Juo P (2015). The DAF-7/TGF-β signaling pathway regulates abundance of the Caenorhabditis elegans glutamate receptor GLR-1.. Mol Cell Neurosci.

[R7] Meisel JD, Panda O, Mahanti P, Schroeder FC, Kim DH (2014). Chemosensation of bacterial secondary metabolites modulates neuroendocrine signaling and behavior of C. elegans.. Cell.

[R8] Riddle DL, Blumenthal T, Meyer BJ, Priess JR. C. Elegans II, Cold Spring Harbor (NY): Cold Spring Harbor Laboratory Press; 1997. Appendix, Genetics; p. 917. 21413221

[R9] Rongo C, Whitfield CW, Rodal A, Kim SK, Kaplan JM (1998). LIN-10 is a shared component of the polarized protein localization pathways in neurons and epithelia.. Cell.

[R10] Thomas JH, Birnby DA, Vowels JJ (1993). Evidence for parallel processing of sensory information controlling dauer formation in Caenorhabditis elegans.. Genetics.

[R11] Trent C, Tsuing N, Horvitz HR (1983). Egg-laying defective mutants of the nematode Caenorhabditis elegans.. Genetics.

[R12] Zheng Y, Brockie PJ, Mellem JE, Madsen DM, Maricq AV (1999). Neuronal control of locomotion in C. elegans is modified by a dominant mutation in the GLR-1 ionotropic glutamate receptor.. Neuron.

